# The combined role of allelic variants of *IRS-1* and *IRS-2* genes in susceptibility to type2 diabetes in the Punjabi Pakistani subjects

**DOI:** 10.1186/s13098-019-0459-1

**Published:** 2019-08-06

**Authors:** Anam Ijaz, Sana Babar, Sumbal Sarwar, Saleem Ullah Shahid, Shabana

**Affiliations:** Department of Microbiology and Molecular Genetics, University of the Punjab, Lahore, Pakistan

**Keywords:** Diabetes mellitus, Polymorphisms, PCR-RFLP, Pakistan

## Abstract

**Background:**

Diabetes mellitus is a multifactorial disorder characterized by a high level of glucose in the blood. Both genetic and environmental factors interact to cause diabetes. Insulin receptor substrate (*IRS*) proteins have a significant part in insulin signaling pathways. We aimed to investigate the relationship of type 2 diabetes with a Gly972Arg (G972R) variant of the *IRS-1* gene and Gly1057Asp (G1057D) polymorphism of *IRS-2* gene in the population of Punjab, Pakistan.

**Methods:**

We collected 926 samples, 500 healthy controls (fasting blood sugar < 99 mg/dL, random blood sugar < 126 mg/dL) and 426 cases with diabetes (fasting blood sugar > 99 mg/dL, random blood sugar > 126 mg/dL). Several anthropometric measurements were measured. Statistical analysis was performed by using SPSS to determine the allele group/genotype frequency of the selected variants in the study population.

**Results:**

The genotyping results of G972R by RLFP-PCR showed the allelic frequency of G = 0.68 and R = 0.32 in controls while G = 0.71 and R = 0.29 in the cases. The minor R allele had a slightly higher frequency in the cases than the controls (OR = 0.86, CI 0.706–1.052, *p* = 0.17). The genotyping results of G1057D showed allelic frequency G = 0.74 and D = 0.26 in the controls while G = 0.961 and D = 0.29 in the cases. The minor D allele appeared to be a risk allele for this SNP although the difference in the allele frequencies was not statistically significant (OR = 1.55, CI 0.961–1.41, *p* = 0.108). The combined genotype analysis showed that the difference in the allele and genotype frequencies reached statistical difference between the cases and the controls as well as the odds ratio substantially increased when the R allele (G972R) was present together with D allele (G1057D) in any combination. When the association of single variants with the lipid traits was observed, only D allele (G1057D) showed significant association with TG, HDL and LDL, however when the analysis was repeated for combined genotypes using general linear model, many more significant associations between the genotype where D allele and R allele are together, were seen with many lipid traits.

**Conclusion:**

In conclusion, the single nucleotide polymorphisms with low-modest effect size may not affect the phenotype individually but when in combination, the effect becomes stronger and more visible, therefore, for the SNP association studies, the more the number of SNPs included in the analysis, the more meaningful the results.

**Electronic supplementary material:**

The online version of this article (10.1186/s13098-019-0459-1) contains supplementary material, which is available to authorized users.

## Background

Diabetes is characterized by chronic hyperglycemia leading to the imbalance of fat, protein and carbohydrate metabolism due to defective insulin action, secretion or both [1]. The prevalence of diabetes is increasing worldwide due to aging, population growth, urbanization, physical inactivity and obesity [2, 3]. The sequelae of diabetes include long term damage and dysfunctioning of various organs leading to their failure [4]. The characteristic symptoms of diabetes mellitus involve polyuria, blurred vision, thirst and weight loss [5, 6]. The persons with either type 1 or type 2 diabetes require insulin treatment at some stage of the disease. Type 2 is the most prevalent form of diabetes that is characterized by insulin resistance [7, 8].

The insulin receptor substrates (IRSs) are the cytoplasmic receptors, important in insulin signaling [9]. They also play a pivotal role in maintaining basic cellular functions like survival, growth and metabolism. After the activation of these proteins, several signaling cascades are activated. This leads to the activation of many downstream effectors. Finally, they gave the insulin signal to branching series of intracellular pathways. There are four members of the IRS family and are similar in their overall architecture [10].

When insulin binds to its receptor on cell surface, IRS-1, a docking protein is phosphorylated and activates phosphatidyl inositol 3-kinase (PI3-kinase). This initiates a cascade of intracellular signaling resulting in a variety of responses by the cell. One of these responses is the activation of GLUT (glucose transport) receptor resulting in increased uptake of glucose by the cell [11]. The individuals with non-insulin dependent diabetes mellitus (NIDDM) have decreased expression and function of IRS-1 in the fat cells. The Gly972Arg polymorphism is the most commonly studied variant of *IRS-1* [12]. Downstream IRS-1, the main docking protein found in the cells is IRS-2 [13]. It acts as the backup protein for the intracellular spread of the insulin signal including the activation of PI3-kinase. However, the hallmark of insulin resistance is that, for the activation of IRS-2, higher concentration of insulin is required. Gly1057Asp (G1057D) is a widely reported variant in *IRS-2* gene whereby glycine (G) is replaced by aspartate (D) at 1057 position in the gene [14, 15]. These SNPs were selected for analysis in Pakistani subjects because (1) Pakistani population represents a unique ethnic group which allows the study of concentrated risk genetic markers (2) the selected SNPs have been investigated in the Caucasians so their analysis can provide the information on the relationship of these genetic variants with daibetes and (3) these SNPs have not been previously investigated in the Pakistani population and the current study is the first report of their study in our population. The biochemical risk factors included were lipid profile parameters namely total cholesterol (TC), very low density lipoprotein (VLDL), LDL-C, high density lipoprotein cholesterol (HDL-C) and triglyceride (TG) levels.

## Methods

### Study subjects

926 subjects (426 diabetic cases and 500 normal controls) were recruited from the local population using a case control design between July 2014 and April 2015. The subject recruitment, inclusion and exclusion criteria have been described previously [3, 16]. Inclusion criteria for diabetic subjects were (i) diabetes diagnosed according to etiologic classification of diabetes by the International Diabetes Federation (IDF) and (ii) confirmation that all the grandparents of the subjects are of Pakistani origin. The exclusion criteria were the presence of any infectious disease, conditions where phlebotomy is contraindicated, age below 10 years, body mass index (BMI) ≤ 18.5 kg/m^2^, pregnancy, handicapped/mentally disturbed individuals, obese (obesity defined as: BMI > 23 kg/m^2^ as overweight and BMI > 26 kg/m^2^ as obese, criteria used for Asian populations as described previously [17], cancer and ethnicity other than Pakistani. The controls were healthy subjects from the general population with normal blood sugar levels ( < 126 mg/dL random or < 99 mg/dL fasting) [16]. All the subjects were genetically unrelated and gave written informed consent. The employed procedures were according to the Helsinki declaration and an ethical approval was obtained from the institutional ethical board.

### Blood sampling

Venous blood was collected from the subjects in the fasting state. 5 mL blood was collected from the median cubital vein using aseptic measures. 2.5 mL of blood was dispensed in an EDTA vial and used for DNA isolation and 2.5 mL was poured in a gel clot activator containing vial and used for biochemical analysis.

### Genetic analyses

The DNA was isolated by manual method. The isolated DNA was stored at 4 °C or at − 20 °C for further use. The presence of DNA was determined by Agarose gel electrophoresis. After isolation, all DNA samples were quantified before amplification using Epoch Biotek microplate reader. The quantification was performed to equalize the final concentration of DNA samples up to approximately 10 ng/μL. The PCR conditions consisted of initial denaturation at 95 °C for 2 min, followed by 35 cycles of denaturation at 95 °C for 30 s, annealing at 57 °C for 45 s for G1057D variant and at 61 °C for 45 s for G972R variant, and extension at 72 °C for 1 min followed by a final extension at 72 °C for 5 min. PCR was done in advanced primus 96 thermal cycler. For Gly972Arg, the sequence of primers was: F: CTTCTGTCAGGTGTCCATCC, R: CTCTGCAGCAATGCCTGTTC. The 311 bp PCR product was digested with the help of *Bst*N1 restriction enzyme and produced 3 fragments of 207 bp, 81 bp and 23 bp. For G1057D variant, the sequence of primers was: F: AGCTCCCCCAAGTCTCCTAA, R: GGCCACACCAAAAGCCATCT. The 291 PCR product was digested using the *Hha*I enzyme and produced 2 fragments one of 268 bp and other of 23 bp. The products were seen by running 2% agarose gel electrophoresis and observing the gel under the U.V light (Additional file 1: Figures S1, S2).

### Statistical analysis

For conducting statistical analysis, MS Excel and SPSS (Statistical Package of Social Sciences) Package were used. Different variables such as Anthropometric, Biochemical and Genotype were analyzed by calculating mean and standard Deviation. Further the study population was tested for Hardy Weinberg Equilibrium (HWE), Chi-square test was also used to test the significance of difference of Allele and Genotype frequencies among diabetic and non-diabetic. To check the relationship of the SNPs with diabetes, *t*-test and logistic regression was conducted. Further the association of Polymorphism with Anthropometric and Biochemical parameters was tested through one way ANOVA. An adjusted *p*-value less than 0.025 was used as a significance cutoff for all such analysis due to inclusion of two SNPs at a time.

## Results

The general characteristics of the subjects have been described elsewhere [16] (Table 1). The study population was tested for Hardy–Weinberg equilibrium by a chi-squared test and was found to be in equilibrium (HWE *p*-value was 0.698 for G972R and was 0.377 for G1057D).

**Table 1:** General characteristics of the study subject

Parameters	Diabetic (n = 426)	Non-diabetic (n = 500)	*p* value
Male	228	272	0.108
Female	198	228	0.108
Age (year)	47.55 ± 12.3	35.78 ± 13.4	0.0001*
Height (ft)	5.50 ± 0.3	5.49 ± 0.59	0.756
Weight (kg)	68.6 ± 13.83	65.38 ± 13.7	0.0004*
BMI (kg/m^2^)	22.7 ± 5.6	21.67 ± 5.3	0.0043*

*BMI* body mass index, *n* total number

* Indicates significant differences

### Allele/genotype frequencies of G972R and G1057D polymorphisms

After SNP genotyping, the genotypic and allelic frequencies were calculated. For Gly972Arg, in controls 230 (46%) individuals had GG genotype, 221 (44.2%) had GR genotype and 49 (9.8%) had RR genotype. Whereas, in cases 226 (53.05%) individuals had GG genotype, 153 (35.91%) had GD genotype and 47 (11.03%) had DD genotype. The odd ratio was 1.83 with 95% confidence interval (CI) 0.98–1.74. The *p*-value was 0.067 which shows an insignificant difference in allele/genotype frequencies between the cases and the controls. Allelic and genotypic frequencies of G1057D polymorphism showed that in controls 270 (54%) individuals had GG genotype, 200 (40.0%) individuals had GD genotype and 30 (6%) individuals had DD genotype. In diabetic subjects 231 (54.2%) individuals had GG genotype, 140 (32.8%) individuals had GD genotype and 55 (12.9%) individuals had DD genotype. The OR ratio of genotypic frequencies was 1.62 and 95% confidence interval was 1.22–2.16. The *p*-value of genotypic frequencies was 0.108 (Table 2). The combined genotype analysis revealed that the coexistence of any combination of the two variants with one or both risk alleles (GG + GD, GG + DD, GR + GD, GR + DD, RR + GD, RR + DD) had significant effect on the outcome as indicated by the odds ratio which increased proportionately when the heterozygous and homozygous genotypes of G972R was analyzes with heterozygous and homozygous genotypes of G1057D (Table 3).

**Table2:** Allele and genotypic frequencies of G972R and G1057D polymorphism

SNP	Genotype/allele	Controls (n = 216)	Cases (n = 249)	OR ratio	95% CI	*p*-value
G972R	GG	230	226	1.83	0.98–1.74	0.067
	GR	221	153			
	RR	49	47			
	R	0.32	0.29	1.49	1.13–1.95	0.0579
	G	0.68	0.71			
G1057D	GG	270	231	1.62	1.22–2.16	0.108
	GD	200	140			
	DD	30	55			
	D	0.26	0.29	1.75	1.35–2.27	0.0006
	G	0.74	0.71			

*OR* odds ratio, *CI* confidence interval

**Table 3:** Diabetes by combined genotype groups

G972R	G1057D	N (controls/cases)	OR (CI), *p*-value
GG	GG	328 (128/200)	0.214 (0.145–0.317), 0.001
	GD	107 (90/17)	
	DD	21 (12/9)	
GR	GG	144 (120/24)	2.128 (1.428–3.170), 0.000
	GD	208 (88/120)	
	DD	22 (13/9)	
RR	GG	29 (22/7)	3.174 (1.730–5.821), 0.000
	GD	25 (22/3)	
	DD	42 (5/37)	

### Effect of the G972R and G1057D polymorphisms on biochemical parameters

Biochemical parameters were checked across the three genotypes for both variants individually by one way ANOVA as well as in combination by multivariate general linear model (GLM). For single variant analysis, G972R did not show statistically significant association with any of the lipid traits. For G1057D variant, the presence of risk allele significantly increased TG and LDL and lowered HDL although there was no effect on TC (Table 4). When the genotypes were analyzed in combination, the number of significant associations increased considerably. It was observed that when the common homozygous heterozygous and risk homozygous genotypes of G972R were examined against common homozygous, heterozygous and risk homozygous genotypes of G1057D. The results of this analysis are shown in Table 5. The table shows that the presence of homozygous risk genotype of G972R in any combination with G1057D (GG + GD, GG + DD, GR + GD, GR + DD, RR + GD, RR + DD) had the greatest effect on the change of lipid parameters (significant rise in TC, TG, LDL-C and a decrease in HDL-C).

**Table 4:** Effect of single variant on lipid parameters

Variant	Genotype	TC	TG	HDL	LDL
G972R	GG	5.04 ± 0.06	2.29 ± 0.03	1.48 ± 0.02	2.42 ± 0.03
	GR	5.01 ± 0.06	2.21 ± 0.03	1.49 ± 0.02	2.41 ± 0.03
	RR	4.96 ± 0.14	2.31 ± 0.07	1.43 ± 0.04	2.45 ± 0.07
	*p*-value	0.877	0.240	0.507	0.897
G1057D	GG	4.97 ± 0.06	2.23 ± 0.03	1.52 ± 0.02	2.39 ± 0.02
	GD	5.06 ± 0.07	2.24 ± 0.04	1.47 ± 0.02	2.43 ± 0.03
	DD	5.12 ± 0.13	2.52 ± 0.07	1.29 ± 0.04	2.58 ± 0.08
	*p*-value	0.531	0.006	0.000	0.039

**Table 5:** Lipid profile parameters by combined genotypes

G972R	G1057D	TC	TG	HDL	LDL
GG	GG	6.19 ± 1.53	2.34 ± 0.84	1.57 ± 0.46	2.27 ± 0.47
	GD	4.57 ± 1.02	2.11 ± 0.71	1.45 ± 0.44	2.42 ± 0.66
	DD	5.06 ± 0.86	2.58 ± 1.29	1.38 ± 0.47	2.27 ± 0.47
GR	GG	4.54 ± 1.14	2.04 ± 0.66	1.66 ± 0.45	2.22 ± 0.48
	GD	5.33 ± 1.43	2.27 ± 0.68	1.50 ± 0.37	2.48 ± 0.76
	DD	4.99 ± 0.78	2.32 ± 0.78	1.37 ± 0.47	2.54 ± 0.64
RR	GG	4.67 ± 1.15	1.98 ± 0.47	1.69 ± 0.43	2.14 ± 0.37
	GD	4.88 ± 1.40	2.16 ± 0.80	1.62 ± 0.47	2.33 ± 0.63
	DD	5.12 ± 1.59	2.62 ± 0.87	1.14 ± 0.33	2.71 ± 0.80
*p-*value		0.001	0.007	0.0001	0.009

## Discussion

The health care burden of diabetes is increasing at an alarming rate globally. The proper management for diabetes is therefore required to lessen its outcomes and to prevent the further complications [18, 19]. The Insulin Receptor Substrates (IRSs) of cytoplasmic adaptor proteins functioning in signal transduction in response to insulin binding at the cell surface consists of IRS-1, IRS-2, IRS-3, and IRS-4, named in order of identification [20, 21]. The identification of *IRS-1* and *IRS-2* gene polymorphisms associated with type2 diabetes was a landmark achievement in the field of diabetes genetics. The Gly972Arg is the most extensively studied polymorphism in *IRS-1* gene, present in a highly conserved region of the protein that is involved in the phosphorylation of the substrate [22]. In an in vitro cell culture study of human mesangial cells, the expression of G972R caused a significant reduction in the insulin-stimulated phosphorylation of IRS-1 and Akt kinase [23]. The association studies of G972R SNP report positive as well as negative findings depending upon sample size, genotyping techniques and ethnicity. A previous study reported that carriers of Gly972Arg are at 25% higher risk of havig diabetes as compared to non carriers [24]. Another study reported the association of Gly972Arg with type 2 diabetes in Mexican population [25]. We found contradictory results compared to the Caucasian Dutch population where Gly972Arg did not appear to play a role in increased risk of type 2 diabetes [26]. However, our results are in accordance with the previous published study to report the association of Gly972Arg with type 2 diabetes [27, 28].

The G1057D variant of *IRS-2* gene has been reported in different groups but results are controversial as well. Certain studies have indicated that this variant is not linked to type 2 diabetes in Chinese, Finish, Turkish and Tunisian populations [29–31]. While some studies have shown that G1057D variant of *IRS-2* gene predisposes mice to type 2 diabetes [32]. Another study reported *IRS-2* polymorphisms as one of the genetic causes of type 2 diabetes [33]. Studies that included individuals from Kurdish Ethnic Group (in West Iran) and Asian Indians reported association of G1057D polymorphism of *IRS-2* gene with type 2 diabetes [34, 35]. Similarly, genetic causes that lead to type 2 diabetes in Iranian and Indian population included G1057D polymorphism [36, 37].

In the current study, we determined the association of the variants of IRS-1 Gly972Arg (G972R) and IRS-2 Gly972Arg (G72R) with diabetes. The genotyping results of G972R showed that the minor R allele had a slightly higher frequency in the cases than the controls. The genotyping results of G1057D showed the minor D allele appeared to be a risk allele for this SNP although the difference in the allele frequencies was not statistically significant. The combined genotype analysis showed that the difference in the allele and genotype frequencies reached statistical difference between the cases and the controls as well as the odds ratio substantially increased when the R allele (G972R) was present together with D allele (G1057D) in any combination. When the association of single variants with the lipid traits was observed, only D allele (G1057D) showed significant association with TG, HDL and LDL, however when the analysis was repeated for combined genotypes using general linear model, many more significant associations between the genotype where D allele and R allele are together, were seen with many lipid traits.

There are certain limitations of our study as the sample size was relatively small and the samples were restricted to the Punjab province only. The mean age of controls and cases is significantly different that may cause bias in the study. There are other genes and polymorphisms that affect this association, but could not be included in the current investigation. However, despite these limitations, the results of the current study demonstrate the role of common variants in the progression of complex diseases like diabetes in the Pakistani population and show that if more variants are included in an investigation, more conclusive results can be obtained about the impact of the genetic markers on the phenotype. To our knowledge, the present study is the first attempt to report the association of IRS variants with diabetes in the local population.

## Conclusion

In conclusion, there are various environmental factors and genes that work together in predisposition of an individual to complex diseases like diabetes. Moreover the single nucleotide polymorphisms can play an important role in the etiology of the complex diseases, however, is important to consider their combined effect on the phenotype.

## Additional file

**Additional file 1: Figure S1.** Amplification of *IRS-1* gene region containing G972R polymorphism. Last well contains a known sized DNA ladder (ThermoScientific SM#0321). **Figure S2.** Amplification of *IRS-2* gene region containing G1057D polymorphism. Last well contains a known sized DNA ladder (ThermoScientific SM#0241).

## Data Availability

All the necessary information has been provided along with the manuscript, however, the corresponding author can be contacted for any information related to this paper.
