# Supplementary material for: The combined role of allelic variants of IRS-1 and IRS-2 genes in susceptibility to type2 diabetes in the Punjabi Pakistani subjects
Source: Diabetol Metab Syndr. 2019 Aug 6;11:64. doi: 10.1186/s13098-019-0459-1 (PMC6683393; doi:10.1186/s13098-019-0459-1)
Supplement: Supplementary file 1 — Additional file 1: Figure S1. Amplification of IRS-1 gene region containing G972R polymorphism. Last well contains a known sized DNA ladder (ThermoScientific SM#0321). Figure S2. Amplification of IRS-2 gene region containing G1057D polymorphism. Last well contains a known sized DNA ladder (ThermoScientific SM#0241). [file 13098_2019_459_MOESM1_ESM.docx]

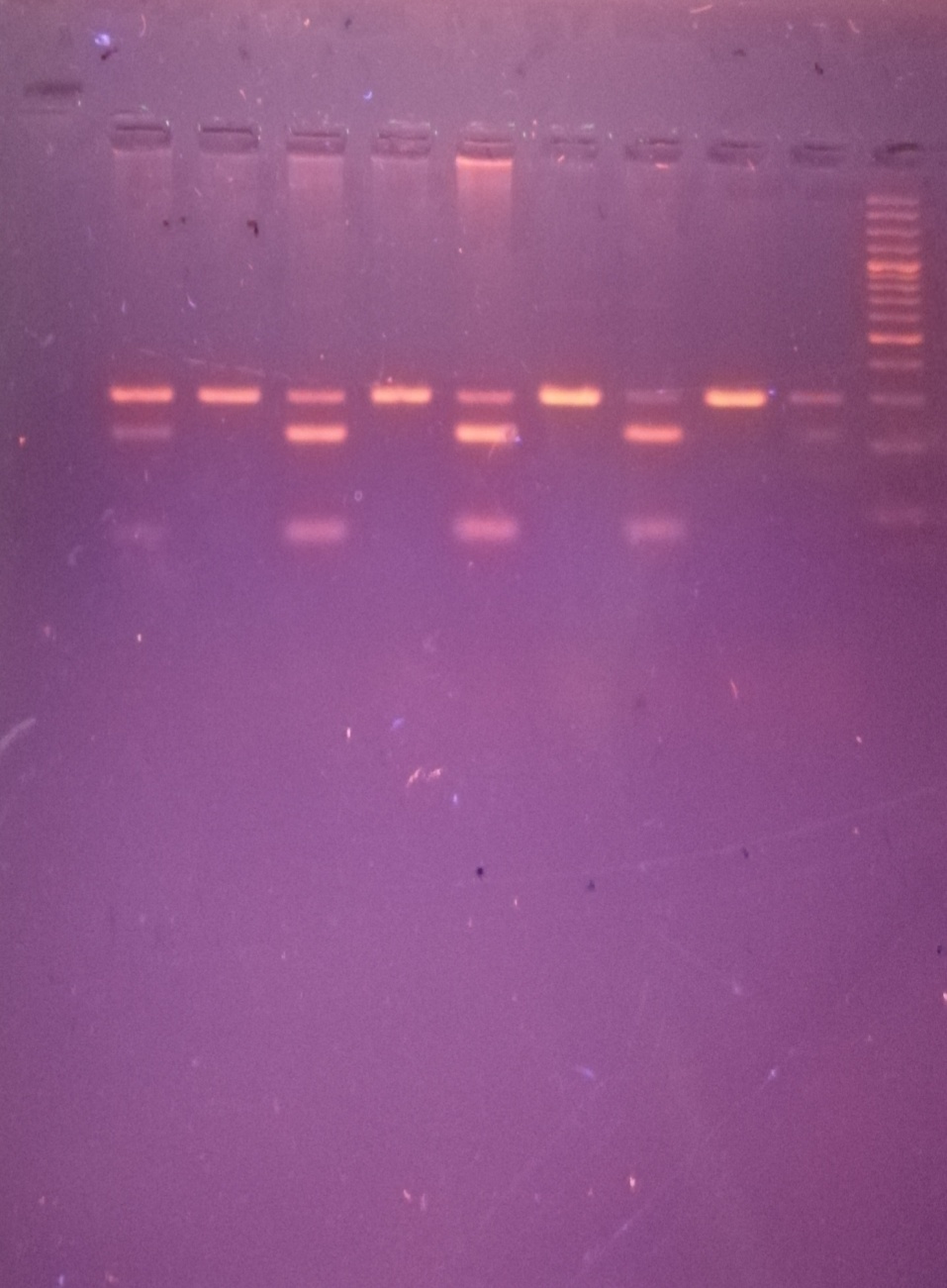


311bp

207bp

81bp

1000bp

500bp

400bp

300bp

200bp

100bp

Figure S1: Amplification of *IRS-1* gene region containing G972R polymorphism. Last well contains a known sized DNA ladder (ThermoScientific SM#0321).

291bp

268bp

23bp


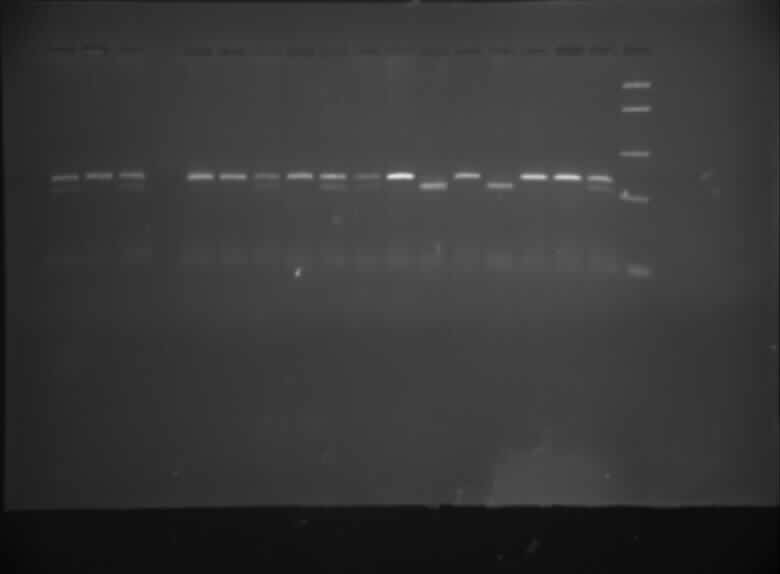


1500bp

800bp

450bp

200bp

50bp

Figure S2: Amplification of *IRS-2* gene region containing G1057D polymorphism. Last well contains a known sized DNA ladder (ThermoScientific SM#0241).
